# Esophageal atresia with tracheo-esophageal fistula

**DOI:** 10.4103/0971-9261.69145

**Published:** 2010

**Authors:** Subir K. Chatterjee

**Affiliations:** Consultant Pediatric Surgeon, Park Clinic, 4, Gorky Terrace, Kolkata 700017, India. E-mail: parkclinic@vsnl.net

Sir,

I read with interest, the recently published paper (1) on esophageal atresia. Many years ago, I had diagnosed esophageal atresia with tracheo-esophageal fistula in the emergency room and had sent the baby to the ward.[1] The resident came running to tell me that he had passed a tube from the nose into the stomach and hence my diagnosis was surely wrong! He had indeed passed a no.6 Fr nasogastric tube; the tube was found to have traversed the nose, the pharynx, the larynx, the trachea, the fistula and the lower esophagus and had reached the stomach.

## References

[1] Hombalkar NN, Dhanawade S, Hombalkar P, Vaze D (2009). Esophageal atresia with tracheo-esophageal fistula: accidental trans-gastric intubation. J Indian Assoc Pediatr Surg.

